# MALT1 Inhibition as a Therapeutic Strategy in T-Cell Acute Lymphoblastic Leukemia by Blocking Notch1-Induced NF-κB Activation

**DOI:** 10.3389/fonc.2020.558339

**Published:** 2020-09-23

**Authors:** Rong Wang, Huihui Zhang, Jiawen Xu, Ninghan Zhang, Ting Pan, Xiaomin Zhong, Huanxin Zhang, Lingling Yin, Yao Yao, Qingyun Wu, Zhenyu Li, Xuejiao Liu, Kailin Xu, Mingshan Niu

**Affiliations:** ^1^Blood Diseases Institute, Affiliated Hospital of Xuzhou Medical University, Xuzhou Medical University, Xuzhou, China; ^2^Department of Hematology, Affiliated Hospital of Xuzhou Medical University, Xuzhou, China; ^3^Department of Medical Oncology, Huai’an First People’s Hospital, Nanjing Medical University, Huai’an, China; ^4^Insititute of Nervous System Diseases, Xuzhou Medical University, Xuzhou, China

**Keywords:** T-ALL, MALT1, MI-2, Notch1, NF-κB

## Abstract

Current treatment of T-cell acute lymphoblastic leukemia (T-ALL) is primarily based on high-intensity combination chemotherapy, which has serious side effects. Therefore, developments of novel targeted therapeutics are urgently needed for treatment of T-ALL. In this study, we found that mucosa-associated lymphoid tissue lymphoma translocation protein 1 (MALT1) is a novel promising therapeutic target for treatment of T-ALL. MALT1 inhibitor MI-2 significantly suppressed the cell growth, proliferation, and colony formation of T-ALL cells. Furthermore, MI-2 induced cell apoptosis of T-ALL via a mitochondrial-dependent pathway. In a T-ALL mouse model, MI-2 significantly reduced leukemic burden and prolonged the survival of leukemia-bearing mice. Mechanistically, MALT1 inhibition effectively blocked both baseline and Notch1-induced activation of nuclear factor κB pathway, which mediates T-ALL cell survival. In conclusion, our results highlight the potential role of MALT1 as an attractive target for treatment of T-ALL and support the potential of MI-2 or other MALT1 inhibitors to clinical trials in T-ALL.

## Introduction

T-cell acute lymphoblastic leukemia (T-ALL) is a genetically heterogeneous disease arising from early T-cell progenitors. It accounts for approximately 15% of pediatric and 25% of adult ALL cases (1). Current treatment is primarily based on high-intensity combination chemotherapy and comes with significant side effects (2). The side effects on bone development and central nervous system in children should not be underestimated (3). In addition to these side effects, the occurrence of relapse is another important challenge. Relapsed T-ALL patients are often associated with chemotherapy resistance and poor prognosis (4). Therefore, developments of novel targeted therapeutics are urgently needed for treatment of T-ALL.

More than 50% of human T-ALLs have activating mutations in Notch1, which was defined as a prominent oncogene for T-ALL (5). Preliminary evidence came from the involvement of chromosomal translocations of Notch1 in patients with T-ALL (6). After three shears, Notch1 generates an intracellular fragment of Notch1 (Notch1-IC), which translocates to the nucleus and exerts its biological functions. Notch1 signaling is central to the differentiation and development of T-cell progenitors (7). Notch1 mutations increase the production of Notch-IC and prolong Notch1-IC half-life (8). Because the primary function of Notch1-IC is to activate transcription, consistently activated Notch1-IC promotes tumorigenesis by effectively multiple tumor-promoting pathways in T-ALL (9).

The nuclear factor κB (NF-κB) pathway is one of the primary downstream targets of oncogenic Notch1 in T-ALL (10). NF-κB is primarily regulated by its inhibitory protein, IκBα, which binds to and sequesters NF-κB in the cytoplasm. Specific stimulation activates the IκB kinase (IKK) complex that phosphorylates IκBα, and then degradation of IκBα allows nuclear translocation of NF-κB and results in the transcription of target genes (11). It was shown that constitutive IKK kinase activity and nuclear localization of NF-κB occur in T-ALL cells (12, 13). Although mutant NF-κB genes have not been reported in T-ALL, constitutive NF-κB activation frequently occurs in T-ALL (14). Furthermore, NF-κB activity in microenvironmental cells also contributes to T-ALL pathogenesis (15). However, the exact molecular mechanism by which Notch1 activates NF-κB in T-ALL remains unclear.

We previously found that CARMA1 is crucial for Notch1-induced NF-κB activation in T-ALL and contributes to Notch1-driven leukemia progression (16). The activated CARMA1 protein can recruit mucosa-associated lymphoid tissue lymphoma translocation protein 1 (MALT1) and BCL10, resulting in formation of the CARMA–BCL10–MALT1 (CBM) complex. MALT1 is the functional component of the CBM complex and features protease activity that cleaves and inactivates inhibitors of the NF-κB pathway. However, no specifically targeted inhibitors of CARMA1 are currently available. MI-2 is an irreversible small-molecule inhibitor of MALT1 (17). It was previously screened as a selective inhibitor of MALT1, which engages and irreversibly binds the active site of MALT1 and suppresses its protease function. MI-2 is effective in preclinical models of activated B cell-like diffuse large B cell lymphoma (ABC-DLBCL). Thus, we asked whether MALT1 inhibition could be a novel therapeutic approach in T-ALL.

In this study, we investigated the potential therapeutic role of MALT1 inhibitor MI-2 in T-ALL. Further, we explored whether MALT1 inhibition could block the Nothch1-induced NF-κB activation in T-ALL.

## Materials and Methods

### Cell Lines and Culture Conditions

T-cell acute lymphoblastic leukemia cell lines CCRF and MOLT-4 were cultured in Iscove’s modified Dulbecco medium supplemented with 10% fetal bovine serum (FBS), 2 mM glutamine, 10 mM HEPES, and penicillin G/streptomycin. 293T cells were cultured in Dulbecco modified eagle medium supplemented with 10% FBS and penicillin G/streptomycin. All cell lines were cultured at 37°C in a humidified atmosphere of 5% CO_2_. CCRF and MOLT4 were transduced with lentivirus expressing Notch1-IC to generate Notch1-IC overexpression cells.

### Cell Viability Assays

For viability assay, CCRF and MOLT-4 were seeded at 1.5 × 10^4^ in 96-well plates and then treated with different concentrations of MI-2 for 24, 48, and 72 h, respectively. Subsequently, 10 μL of Cell Counting Kit-8 (CCK-8) was added to each well. After incubation for 2 h, the absorbance was measured at a wavelength of 450 nm.

### Colony Formation Assay

For colony formation assay, CCRF and MOLT-4 cells were seeded at a density of 1,000 cells/well in six-well plates, and cells were treated with different concentrations of MI-2 one time. The medium contains 0.9% methylcellulose (Sigma-Aldrich, St. Louis, MO, United States), 20% FBS (Gibco), and the different dose of MI-2. After incubation for 12 days, the numbers of colonies were counted manually under the microscope. The images of colonies were also observed and pictured at 40× magnification, using an IX71 inverted microscope (Olympus, Tokyo, Japan).

### EdU Assay

The proliferative ability was detected using kFlour647 Click-iT EdU Flow Detection Kit (KeyGEN BioTECH). According to the manufacturer’s instruction, cells were incubated with 20 μM EdU (5-ethynyl-2′-deoxyuridine) for 4 h after drug exposure. Subsequently, the cells were washed twice with phosphate-buffered saline (PBS) and then treated with fixation and permeabilization buffer (BD Biosciences) for 30 min. After one wash with PBS, the cells were incubated with 1× buffer for 30 min. After one wash with PBS again, the cells were incubated with click-i7 buffer for 30 min in the dark. Followed by two washes with 1× buffer, then cell pellets were resuspended in PBS. The EdU-positive rate was measured by flow cytometry and was analyzed by the FlowJo program (version 10; Ashland, OR, United States).

### Caspase-Glo 3/7 and Caspase-Glo 9 Assay

CCRF and MOLT-4 were seeded in 96-well plates and treated with MI-2 for 12 h. Caspase-Glo 3/7 and caspase-Glo 9 enzymatic activities were measured according to the manufacturer’s protocol (Promega); 100 μL of the reagent was added to samples, and the solution was gently mixed. After incubation for 30 min, 200 μL of solution was transferred into white-walled multiwell luminometer plates, and the luminescence of each sample was measured by GloMax Luminometer (Promega).

### Apoptosis Assay

Fluorescein isothiocyanate (FITC) annexin V Apoptosis Detection Kit I (BD Biosciences) was used to identify the cell apoptosis according to the manufacturer’s instructions. Briefly, cells were seeded into six-well plates at 10^6^ cells per well. MI-2 was added at a concentration series ranging from 0.5 to 2 μM. After 12/24 h of culture, cells were washed twice with cold PBS, resuspended in binding buffer, and stained with FITC annexin V and propidium iodide. Finally, the apoptosis assay was evaluated by fluorescence-activated cell sorting within an hour.

### Western Blot Analysis

Western blot analysis was conducted as described previously (18, 19). Cell lysis buffer (Cell Signaling Technology) was used to extract total protein from the cultured cells. Subsequently, protein concentration was determined by the Enhanced BCA Protein Assay kit (Beyotime Institute of Biotechnology). Proteins were separated by sodium dodecyl sulfate–polyacrylamide gel electrophoresis and transferred onto polyvinylidene fluoride membranes. Membranes were blocked for 2 h and incubated overnight at 4°C with primary antibody. Then membranes were washed, incubated with appropriate secondary antibodies for 1 h, and detected with ECL substrate (Bio-Rad). Primary antibodies against actin and Cdc2-p34, IκBα were obtained from Santa Cruz Biotechnology (Santa Cruz, CA, United States), and antibodies against p21, p27, cdc25C, PARP, survivin, Bcl-xL, Bcl-2, Bax, p65, and H2A were purchased from Cell Signaling Technology (Danvers, MA, United States).

### Dual-Luciferase Reporter Assay

Reporter assays were performed in 293T cells seeded at a density of 2 × 10^5^ cells per well in a six-well dish. Notch-IC overexpression vector, NF-κB-Luc reporter vector, and pRL-TK internal control plasmids were cotransfected using TurboFect Transfection Reagent (Thermo Fisher Scientific). In CCRF and MOLT-4 cells stably expressing Notch1-IC, NF-κB-Luc reporter vector and pRL-TK internal control plasmid were cotransfected using electroporation. Twelve hours after transfection, cells were treated with different concentrations of MI-2. Lysates were submitted to Dual-Glo Luciferase Assay System following the manufacturer’s protocol (Promega).

### Mouse Xenograft Experiments

Five-week-old male NSG mice were purchased from Vital River Laboratories. All animal experimental protocols were approved by the ethics committee of the Xuzhou Medical University. For the T-ALL mouse model, 5 × 10^4^ CCRF cells were injected into the tail of NSG mice. After 5 days, MI-2 (20 mg/kg per day) and dimethyl sulfoxide vehicle were intraperitoneally injected into the mice for 5 days every week. One day after the 4-week treatment, mice were randomly picked up (three mice per group), and then peripheral blood (PB), bone marrow (BM), spleen, and liver samples were collected for further analysis.

### Flow Cytometric Analysis

To assess the level of leukemia burden in the mice transplanted with CCRF, cells from BM, spleen, liver, and PB were lysed with ACK Lysing Buffer (Gibco) and then buffered with PBS and 1% FBS before analysis. Flow cytometry quantification of hCD45^+^ cells in CCRF xenograft models was performed using an anti–human CD45 antibody (BD Biosciences). All data were analyzed with FlowJo program (version 10).

### H&E Staining and Immunohistochemistry

Representative tissues of liver, spleen, and BM were fixed with 10% formaldehyde buffer for over 24 h. After dehydration, tissues were embedded in paraffin, and sections were cut. Hematoxylin-eosin staining was performed on deparaffinized sections. For immunohistochemistry, the sections were blocked and incubated with primary antibody CD45 (Cell Signaling Technology). The color was developed with diaminobenzidine and counterstained with hematoxylin (20). For BM sections, after fixation and decalcification, paraffin-embedded slides were deparaffinized and rehydrated. The slides were immersed in citrate antigen retrieval solution and 3% H_2_O_2_ for 25 min. Subsequently, the slides were blocked with 3% bovine serum albumin for 30 min and incubated with anti-CD45 primary antibody (Cell Signaling Technology) overnight at 4°C. The slides were washed with PBS and incubated with secondary antibodies for 50 min. Then the slides were developed with DAB chromogenic reagent and counterstained with hematoxylin staining solution for 3 min and washed in tap water. Finally, slides were dehydrated in ascending alcohol concentrations and cleared with xylene.

### Statistical Analysis

The results are expressed as the mean ± SD of three independent experiments. Levels of significance were evaluated by Student *t-*test, using the GraphPad Prism software. All tests were performed as two-sided, and *P* < 0.05 was considered statistically significant.

## Results

### MALT1 Inhibitor MI-2 Suppresses the Cell Growth of T-ALL

**FIGURE 1:**
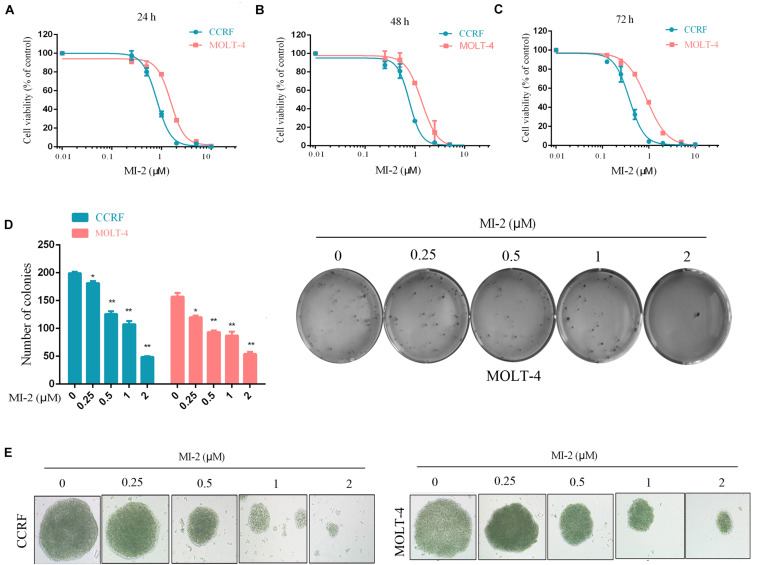
MI-2 inhibited the growth of T-ALL cells. **(A–C)** CCRF and MOLT-4 were incubated with increasing concentrations of MI-2 for 24, 48, and 72 h, respectively. Cell viability was quantified using CCK-8 assay and shown as% of the untreated control. **(D,E)** CCRF and MOLT-4 were cultured in methylcellulose with indicated doses of MI-2 for 12 days. MI-2 significantly decreased the quantity and size of cell colonies. *P < 0.05, **P < 0.01.

To examine the effect of MI-2 on T-ALL cell growth, we evaluated the viability of CCRF and MOLT-4 cells treated with MI-2 using the CCK-8 assay. We found that MI-2 could markedly inhibit the growth of CCRF and MOLT-4 at micromolar concentrations (Figures 1A–C). MI-2 inhibited CCRF and MOLT-4 cells in dose-dependent and time-dependent manners. CCRF seems more sensitive to MI-2 compared with MOLT-4 at the same concentration. The colony formation assay was conducted to evaluate the long-term effects of MI-2 on clonal proliferation. As shown in Figures 1D,E, the quantity and size of cell colonies were markedly decreased after a 12-day exposure to MI-2. Taken together, these results demonstrate that MI-2 is preferentially cytotoxic for T-ALL cells.

### MI-2 Inhibits the Cell Proliferation and Regulates the Expression of Cell Cycle Regulators

**FIGURE 2:**
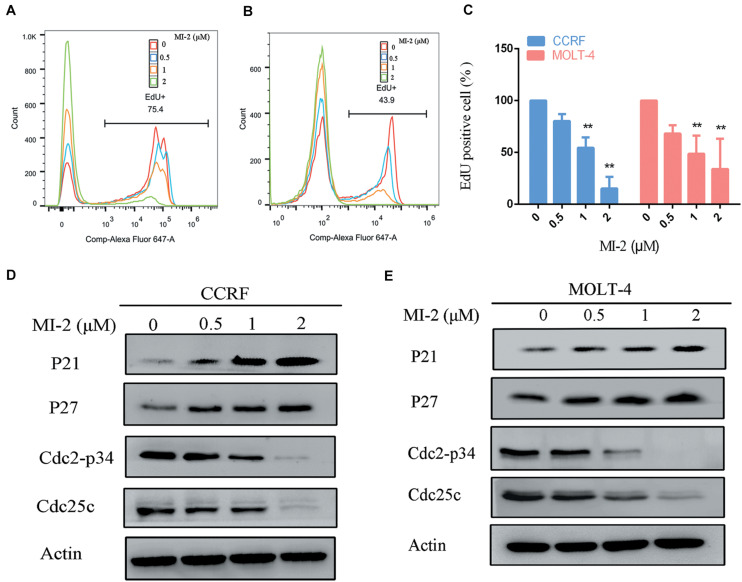
MALT1 is associated with the proliferation of T-ALL cells. **(A,B)** The effect of MI-2 on proliferation was determined by FACS after MI-2 treatment. **(C)** The percentage of EdU positive cells was quantified in CCRF and MOLT-4. CCRF and MOLT-4 cells were treated for 12 h. **(D,E)** Expression of cell cycle related proteins was evaluated by western blot. **P < 0.01.

To confirm the efficacy of MI-2 in T-ALL cells, we tested cell proliferation using EdU assay. As shown in Figures 2A–C, MI-2 significantly reduced the proliferation of CCRF and MOLT-4 cells. The rates of EdU positive cells in CCRF and MOLT-4 were reduced prominently with the increment of MI-2. To investigate whether the repressed cell proliferation was caused by the arrest of cell cycle progression, western blot analysis was conducted to explore the effects of MI-2 on cell cycle–related molecules. The results confirmed MI-2 induced cell cycle arrest as evidenced by upregulation of p21 and p27. The levels of mitosis-related molecules Cdc2-p34 and Cdc25c were potently downregulated (Figures 2D,E). These results confirm that MI-2 inhibits cell proliferation of T-ALL by modulating multiple cell cycle regulatory proteins.

### MALT1 Inhibition Induces Apoptosis of T-ALL Cells

**FIGURE 3:**
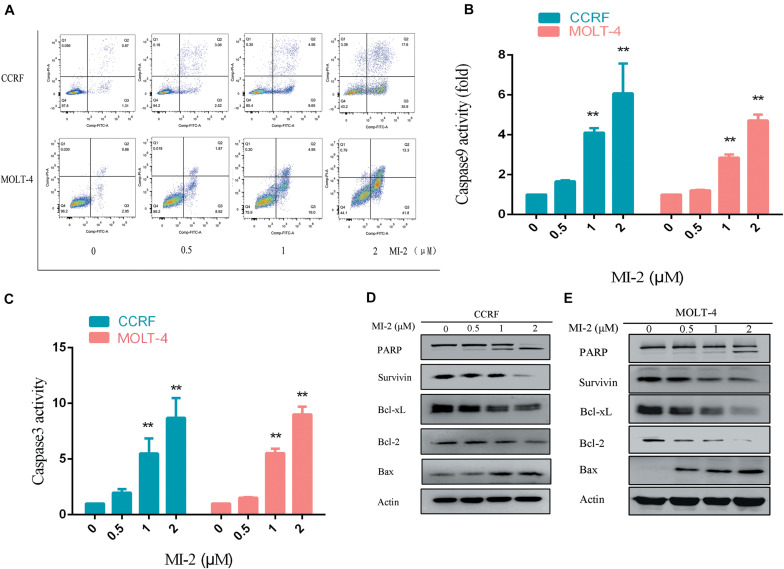
MI-2 induced apoptosis of T-ALL cells in a dose-dependent manner. **(A)** The effect of MI-2 on cell apoptosis was analyzed by FACS. **(B,C)** CCRF and MOLT-4 were plated with the indicated concentration of MI-2 for 12 h. Caspase-9 and caspase-3/7 activity were assessed according to the manufacturer’s protocol. **(D,E)** Representative images of apoptosis-related protein were shown. **P < 0.01.

To determine the effect of MI-2 on T-ALL cell apoptosis, the CCRF and MOLT-4 cells were treated with MI-2, followed by a flow cytometry analysis to assess cell apoptosis. The percentage of apoptotic cells was significantly increased in MI-2 treated groups in a concentration-dependent manner, compared to control group (Figure 3A). Caspases are a family of cysteine proteases that serve as primary effectors during cell apoptosis. As shown in Figures 3B,C, MI-2 treatment significantly activated caspase-9 and caspase-3/7. To investigate the molecular mechanism of apoptosis, we detected Bcl-2 family proteins, PARP and survivin, using western blot analysis (Figures 3D,E). Treatment with MI-2 induced the cleavage of PARP; decreased the expression of survivin, Bcl-xL, and Bcl-2 proteins; and increased the expression of Bax proteins in CCRF and MOLT-4 cells. Taken together, these results suggested that MI-2 induces apoptosis of T-ALL cells via the mitochondrial-dependent pathway.

### MI-2 Treatment Is Effective in a Mouse Xenograft Model of T-ALL

**FIGURE 4:**
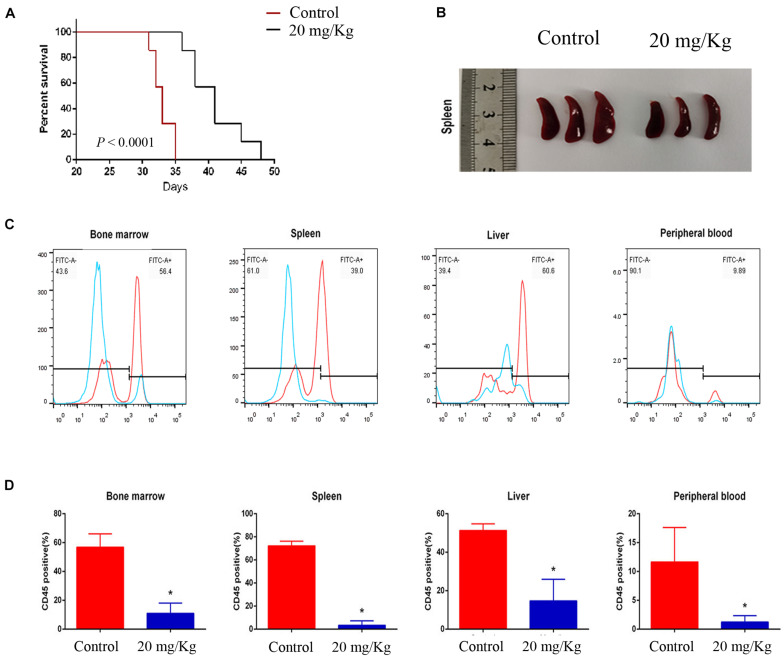
MI-2 impaired leukemia progression *in vivo*. **(A)** Kaplan–Meier survival curves for recipient mice transplanted with CCRF leukemic cells are shown. Administration of vehicle or MI-2 (20 mg/kg per day for 20 days) began 2 days after transplantation. **(B)** Representative photographs of differences in spleen sizes are shown. **(C)** FACS analysis of the distribution of human CCRF cells in BM, PB, spleen, and liver of mice and stained with anti-human CD45. **(D)** The percentage of CD45 positive cells was quantified. *P < 0.05.

**FIGURE 5:**
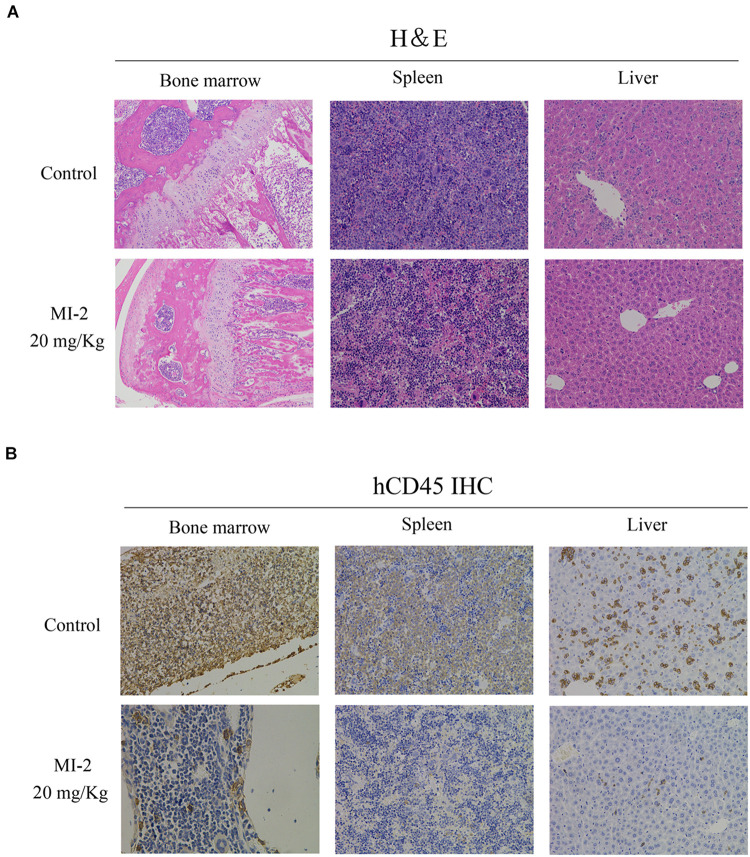
The pathological morphologies of mouse tissues. **(A)** Hematoxylin-eosin staining for the indicated tissues in animals treated with control or MI-2. **(B)** Immunohistochemistry staining of CD45 positive cell in the BM, spleen and liver of control and MI-2 group.

To evaluate the preclinical efficacy of MI-2 *in vivo*, we used a mouse xenograft model of T-ALL. We found that MI-2 treatment significantly retarded disease progression. We observed an increased overall survival in MI-2 treatment group compared to the control group (Figure 4A). We also observed a significant reduction in spleen size by MI-2 treatment (Figure 4B). From the flow cytometry analysis, MI-2 significantly inhibited the rate of hCD45-positive cells in PB, BM, spleen, and liver (Figures 4C,D). The pathological morphologies showed that MI-2 remarkably suppressed the tissue infiltration and reduced the percentage of hCD45-positive T-ALL cells in BM, spleen, and liver (Figures 5A,B). These data suggested that MI-2 exhibits significant activity against disease in T-ALL mouse model and is sufficient to potently impair T-ALL progression *in vivo*.

### MALT1 Inhibition Blocks Notch1-Induced NF-κB Activation in T-ALL Cells

**FIGURE 6:**
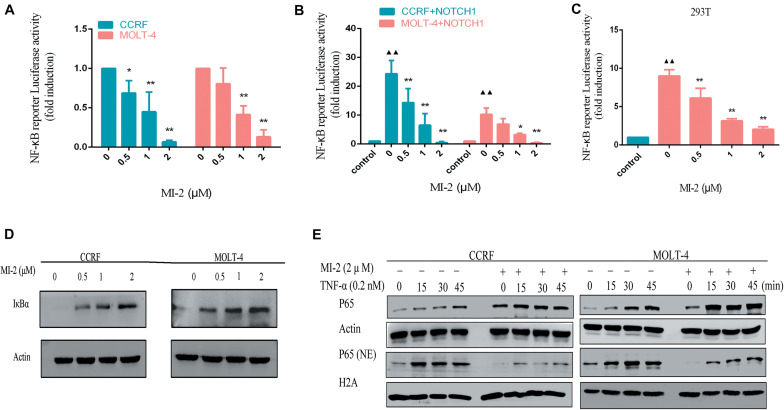
MALT1 inhibition abolished Notch1-induced NF-κB activation. **(A)** NF-κB reporter assays were performed in CCRF and MOLT-4 cells treated with indicated concentrations of MI-2. **(B)** Exogenous Notch1 significantly upregulated tNF-κB activity. NF-κB reporter assays were performed in CCRF and MOLT-4 stably expressing Notch1-IC treated with indicated concentrations of MI-2. The Notch1-IC-induced luciferase activity data were normalized to empty vector-transfected cells. **(C)** NF-κB reporter assays were conducted in 293T stably expressing Notch1-IC. Cells were treated with indicated concentrations of MI-2. The Notch1-IC-induced luciferase activity data were normalized to empty vector-transfected 293T cells. **(D)** The effect of MI-2 on IκBα expression in CCRF and MOLT-4 is shown. **(E)** Nuclear and cytoplasmic expression of NF-κB subunits p65 were assessed by western blot following exposure to MI-2 and TNF-α. *P < 0.05, **P < 0.01.

To determine whether MALT1 is responsible for promoting Notch1-induced NF-κB activity in T-ALL cells, we measured the activation of NF-κB in T-ALL cells. With the increasing concentrations of MI-2, the activity of NF-κB in CCRF and MOLT-4 was potently decreased (Figure 6A). Then, we infected CCRF and MOLT-4 cells with a lentivirus expressing Notch1-IC. We found that exogenous expression of Notch1-IC effectively activated NF-κB, whereas NF-κB activity was partially blocked by MALT1 inhibition (Figure 6B). The same results were also observed in 293T (Figure 6C). Thus, MALT1 might play an essential role in Notch1-induced NF-κB activation, and MI-2 treatment could inhibit the activity of NF-κB in T-ALL.

Numerous negative regulators regulate the activation of NF-κB pathway and function as tumor suppressors. Among them, IκBα is identified as a major brake on NF-κB pathway (21). As shown in Figure 6D, MI-2 treatment significantly increased the expression of IκBα in a dose-dependent manner. Aberrant NF-κB activation underlies the development of many cancers. Inflammatory cytokines abundant in cancer, including tumor necrosis factor α (TNF-α), have been shown to activate NF-κB constitutively in tumor cells (22). We found that MALT1 inhibition significantly prevented the TNF-α–induced p65 nuclear translocation both in CCRF and MOLT-4 cells (Figure 6E). Taken together, MALT1 might play an essential role in Notch1-induced NF-κB activation, and MI-2 treatment could inhibit the activity of NF-κB in T-ALL.

## Discussion

**FIGURE 7:**
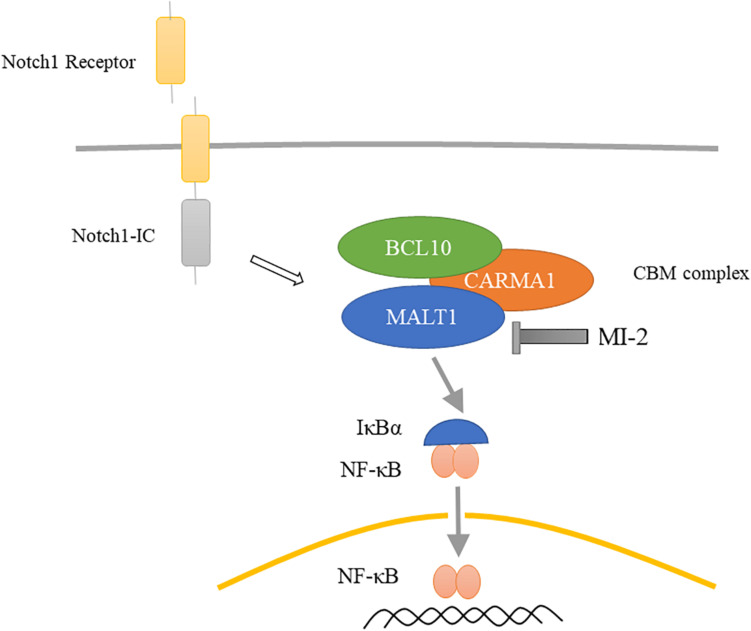
Proposed mechanism for MALT1 inhibition by MI-2. MALT1 is crucial for Notch1-induced NF-κB activation. MI-2 inhibits NF-κB activation by increasing IκBα proteins and inhibiting p65 nucleus translocation.

The current treatment of T-ALL consists of high-intensity combination chemotherapy, which has acute toxicity and long-term side effects. Hence, identifying novel targets for the development of specific and less detrimental therapies for T-ALL is urgently needed. In this study, we demonstrated that MALT1 is a promising therapeutic target, and MI-2 is efficacious for T-ALL treatment. We observed that MALT1 contributes to the survival of T-ALL cells and mediates Notch1-induced NF-κB signaling (Figure 7). Additionally, MI-2 is non-toxic to mice and significantly suppressed the growth of T-ALL cells *in vivo*.

Notch1 is crucial for the commitment to the lymphoid lineage and T-cell development (23). Deregulated Notch1 signaling is associated with T-ALL leukemogenesis. Current approaches including antibodies against the Notch receptor or ligand and inhibition of γ-secretase are in various stages of testing. However, off-target effects and severe diarrhea restrict further development. Given these disadvantages, it seems that targeting the component of Notch1 signaling pathway might be a promising treatment strategy. Notch1 is essential for activating NF-κB pathway, which is a potential therapeutic target in many tumors. However, NF-κB inhibitions have not been successfully introduced to clinical tests because of the important function of NF-κB pathway in many normal cells. Thus, it is necessary to explore tumor-specific inhibitor targeting NF-κB. We previously found that CARMA1 is specifically highly expressed in T-ALL and mediates Notch1-induced NF-κB activation, while there is no inhibitor targeted CARMA1 (16). CARMA1-BCL10-MALT1 complex plays a critical role in NF-κB pathway. In this study, MALT1 was found to contribute to T-ALL progression and is involved in Notch1-induced NF-κB activation. We speculate that targeting MALT1 might be a propelling therapeutic regimen.

MI-2 is an irreversible inhibitor of MALT1, which has been reported effective in chronic lymphocytic leukemia and ABC-DLBCL (17, 24). In this study, we observed that MI-2 attenuated proliferation and induced apoptosis of T-ALL cells. MI-2 also significantly prolonged the survival of T-ALL xenograft models. Dual-luciferase reporter assay suggests that MI-2 suppressed the NF-κB activity and MALT1 might mediate Notch1-induced NF-κB signaling. MI-2 also inhibited the expression of NF-κB target gene proteins, as shown by the decreased expression of Bcl-2 and Bcl-XL. Furthermore, MI-2 increased IκBα expression and inhibited p65 nucleus translocation. In brief, MALT1 represents an attractive therapeutic target for T-ALL.

The human MALT1 gene was initially discovered through the study of chromosomal translocation in MALT lymphomas. The chromosomal translocation creates the cIAP-MALT1 fusion protein that constitutively promotes NF-κB activation and contributes to the malignancy of MALT lymphoma (25). MALT1 is the only human gene encoding paracaspase. It shares homology with caspases because of the caspase-like domain with conserved catalytic cysteine and histidine residues (26). Besides the caspase-like domain, MALT1 comprises an N-terminal death domain followed by two immunoglobulin-like domains and a third, immunoglobulin-like domain located at the C-terminal (27). It was reported that MALT1-deficient mice primarily impinge on lymphocyte function and activation but appeared to be healthy (28, 29). Collectively, these factors suggest that specific inhibitors targeting MALT1 might be prospective candidates for T-ALL with limited toxicity.

## Conclusion

In summary, our results demonstrate that MALT1 inhibitor MI-2 can significantly inhibit the proliferation of T-ALL cells *in vitro* and *in vivo*. Notably, MALT inhibition effectively blocks both baseline and Notch1-induced activation of NF-κB pathway mediating T-ALL cell survival. These findings suggest that MALT1 may be a novel promising therapeutic target for T-ALL treatment and support the potential of MI-2 or other MALT1 inhibitors to clinical trials in T-ALL.

## Data Availability Statement

The raw data supporting the conclusions of this article will be made available by the authors, without undue reservation.

## Ethics Statement

The animal study was reviewed and approved by the Ethics Committee of the Xuzhou Medical University.

## Author Contributions

RW, HHZ, and JX performed the main experimental procedures. NZ, TP, XZ, HXZ, LY, and YY participated in partial experiments. QW and ZL participated in statistical analysis. XL, KX, and MN contributed to study design and drafted the manuscript. All the authors read and approved the final manuscript.

## Conflict of Interest

The authors declare that the research was conducted in the absence of any commercial or financial relationships that could be construed as a potential conflict of interest.

## Abbreviations

CBM: CARMA–BCL10–MALT1
MALT1: mucosa-associated lymphoid tissue lymphoma translocation protein 1
Notch1-IC: intracellular fragment of Notch1
T-ALL: T-cell acute lymphoblastic leukemia.
